# Increased Functional Activation of Limbic Brain Regions during Negative Emotional Processing in Migraine

**DOI:** 10.3389/fnhum.2016.00366

**Published:** 2016-07-26

**Authors:** Sophie L. Wilcox, Rosanna Veggeberg, Jordan Lemme, Duncan J. Hodkinson, Steven Scrivani, Rami Burstein, Lino Becerra, David Borsook

**Affiliations:** ^1^Center for Pain and the Brain (PAIN Research Group), Boston Children’s Hospital, Harvard Medical School, BostonMA, USA; ^2^Department of Anesthesiology, Perioperative and Pain Medicine, Boston Children’s Hospital, Harvard Medical School, BostonMA, USA; ^3^Department of Psychiatry, PAIN Research Group, Brain Imaging Center, McLean Hospital, Harvard Medical School, BelmontMA, USA; ^4^Department of Oral and Maxillofacial Surgery, Massachusetts General Hospital, BostonMA, USA; ^5^Anesthesia, Critical Care and Pain Medicine, Beth Israel Deaconess Medical Center, Harvard Medical School, BostonMA, USA

**Keywords:** migraine, headache, fMRI, emotion, IAPS, limbic

## Abstract

Pain is both an unpleasant sensory and emotional experience. This is highly relevant in migraine where cortical hyperexcitability in response to sensory stimuli (including pain, light, and sound) has been extensively reported. However, migraine may feature a more general enhanced response to aversive stimuli rather than being sensory-specific. To this end we used functional magnetic resonance imaging to assess neural activation in migraineurs interictaly in response to emotional visual stimuli from the International Affective Picture System. Migraineurs, compared to healthy controls, demonstrated increased neural activity in response to negative emotional stimuli. Most notably in regions overlapping in their involvement in both nociceptive and emotional processing including the posterior cingulate, caudate, amygdala, and thalamus (cluster corrected, *p* < 0.01). In contrast, migraineurs and healthy controls displayed no and minimal differences in response to positive and neutral emotional stimuli, respectively. These findings support the notion that migraine may feature more generalized altered cerebral processing of aversive/negative stimuli, rather than exclusively to sensory stimuli. A generalized hypersensitivity to aversive stimuli may be an inherent feature of migraine, or a consequential alteration developed over the duration of the disease. This proposed cortical-limbic hypersensitivity may form an important part of the migraine pathophysiology, including psychological comorbidity, and may represent an innate sensitivity to aversive stimuli that underpins attack triggers, attack persistence and (potentially) gradual headache chronification.

## Introduction

Pain, by its definition, is comprised of both “an unpleasant sensory and emotional experience” (Merskey and Bogduk, 1994). This is evident in pain conditions, such as migraine, where emotional factors and headache symptoms have been demonstrated to have a complex, bidirectional relationship (Spierings et al., 1997; Lanteri-Minet et al., 2005; Walters et al., 2014). There is a higher co-occurrence of emotional disturbances including anxiety, depression, phobias and panic disorders in migraineurs, compared to the general population (Radat and Swendsen, 2005). Furthermore, heightened emotion (in particular stress) is the most commonly reported trigger for migraine attacks (Kelman, 2007).

The relationship between pain and emotion is also highlighted by the overlap in their central representation in the brain. Pain (Peyron et al., 2000; Duerden and Albanese, 2013) and emotion (Phan et al., 2002) both activate a wide array of cortical and subcortical regions, overlapping in areas including the insula, cingulate, thalamus, amygdala, and caudate (Vogt, 2005; Cauda et al., 2012). This neuroanatomical overlap is highly relevant given that one pathophysiologic mechanism of migraine is an altered cerebral processing of sensory stimuli (including pain (Burstein et al., 2010; Moulton et al., 2011), light (Boulloche et al., 2010; Bjork et al., 2011; Denuelle et al., 2011; Martin et al., 2011; Cucchiara et al., 2015) and smell (Demarquay et al., 2008; Stankewitz and May, 2011)) attributed to enhanced cortical excitability and/or dishabituation (Coppola et al., 2007; Brighina et al., 2009; Goadsby et al., 2009). However, as previously mentioned, pain is essentially a negative emotional experience. In the context of migraine, where patients may report abnormal sensory tolerances to light (photophobia) (Vanagaite et al., 1997; Mulleners et al., 2001), sound (phonophobia) (Rojahn and Gerhards, 1986; Vingen et al., 1998; Ashkenazi et al., 2009) and smell (Demarquay et al., 2006), these sensory stimuli may also have a significant negative emotional component (Rojahn and Gerhards, 1986). This raises the potential that migraine may feature altered cerebral processing of aversive stimuli in general, rather than sensory specific.

In support of this view a limited number of studies have shown altered processing of emotional stimuli in migraine, specifically in regards to negative or aversive emotion. Andreatta et al. (2012) showed that migraineurs respond to angry, but not neutral or happy, facial expressions preferentially and more intensively than healthy individuals. In relation, de Tommaso et al. (2009) showed that affective images are able to modulate pain perception and cortical response in migraineurs, whereas other modalities of distraction (i.e., mental arithmetic) were not effective.

The purpose of the present study was to assess the brain correlates of emotional processing in patients with episodic migraine during the interictal period using functional Magnetic Resonance Imaging (fMRI). We hypothesized we would observe enhanced neural activation in migraineurs in areas involved in emotional and pain processing (i.e., amygdala, hippocampus, prefrontal cortex, anterior cingulate gyrus) in response to viewing negative pictures. In contrast, we expected no differences between migraineurs and controls in neuronal response to positive and neutral emotional stimuli.

## Materials and Methods

### Subjects

The data from forty-six age- and gender-matched subjects (*n* = 23 migraine patients and *n* = 23 healthy control subjects) who underwent an imaging session at McLean Hospital were included in this study. All migraine patients fulfilled the International Classification for Headache II criteria for episodic migraine (confirmed by a neurologist) and, as part of the inclusion criteria, suffered from migraines for more than 3 years. Clinical characteristics (including medication usage) for individual migraine subjects are given in **Supplementary Table S1**. Migraine patients were scanned during their interictal (headache-free) period, defined as migraine free at least 48 h before and 24 h after the imaging session. Healthy control subjects had no significant history of migraine, or other headache condition. All subjects were screened for depression [Beck Depression Inventory II (Beck et al., 1996)], exclusionary score >25 (moderate to severe depression) and had no history of any other chronic pain, psychiatric (including clinical depression and/or anxiety) or neurological disorder, or any other major disease. Subjects were also screened by a urine test for barbiturates, benzodiazepines, amphetamine, cocaine, tetrahydrocannabinol, phencyclidine, and opioids (excluding prescription pain medications). The study protocol was approved by the Institutional Review Board at McLean Hospital and was conducted in accordance with the principles of the Declaration of Helsinki. Informed consent was obtained from all subjects prior to participation in the study.

### Emotional Stimuli and Scanning Paradigm

**FIGURE 1:**
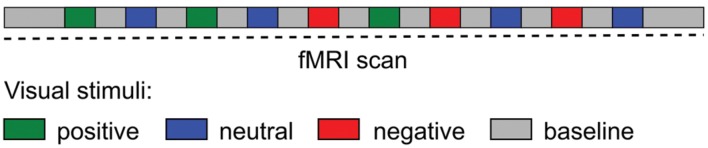
**Schematic diagram of the experimental paradigm during the fMRI scan.** Visual stimuli (International Affective Picture System) blocks were presented in a pseudo-randomized order.

Emotional visual stimuli consisted of positive (pleasant), neutral and negative (unpleasant) color photographs selected from the International Affective Picture System (IAPS; Lang et al., 2008). The IAPS is a standardized, emotionally evocative visual stimulus that has been widely used in studies of emotion, including functional imaging (e.g., de Tommaso et al., 2009; Kamping et al., 2013; Neta et al., 2013; Boucher et al., 2015). A total of 100 IAPS images were displayed (30 positive, 30 negative, and 40 neutral images). The baseline visual stimuli consisted of a black cross hair presented in the middle of a gray screen, a control condition used in other fMRI studies of emotion (Gur et al., 2002; Aldhafeeri et al., 2012). The experimental paradigm presented in the scanner consisted of 10 blocks of emotional visual stimuli with duration for each block of 25 s followed by the baseline visual stimuli with duration of 30 s, with the exception of the first and last baseline with duration of 1 min. During each emotional visual block 10 IAPS images were presented for 2.5 s each. Subjects were not aware of the presentation order and emotional-visual blocks were presented in the same pseudo-randomized order for all subjects (**Figure 1**). The timing of the visual stimuli was programmed using Microsoft PowerPoint. Subjects lay in the MRI scanner and viewed, via a mirror affixed to the head coil, visual stimuli projected onto a screen placed behind the scanner. Before the paradigm began subjects were instructed to keep their eyes open throughout the scan, to blink as normal and to fixate on the cross hair during the baseline visual stimuli. After the imaging session subjects were reshown the presentation and asked to rate each stimulus block for their average emotional valence and arousal using the Self-assessment manikin (SAM) scale (Bradley and Lang, 1994). The SAM scale is an affective rating system that uses graphic figures (manikins) to depict emotional reactions. Subjects were instructed to select any of the nine sequential figures comprising each scale, which resulted in a 9-point rating scale for valence and arousal. Ratings were scored such that a higher score represents a high rating on each scale.

### fMRI Data Acquisition

MRI data were collected on a 3 Tesla Siemens Trio scanner with a 12-channel phased array head coil (Erlangen, Germany). fMRI data were collected using a gradient echo-echo planar pulse sequence with 3.5 mm × 3.5 mm × 3.5 mm resolution. fMRI scan parameters: time of Repetition = 2500 ms, Time of Echo = 30 ms, Field of View = 224 × 224, Flip Angle = 90°, # of Slices = 41 axial slices, # of Volumes = 256. Magnetization-Prepared Rapid Acquisition Gradient-Echo anatomical images were also collected. Anatomical scan parameters: time of Repetition = 2000 ms, Time of Echo = 3.53 ms, Time of Inversion = 1100 ms, Flip Angle = 8°, 224 sagittal slices.

### fMRI Data Preprocessing and Analysis

Functional magnetic resonance imaging data processing and analysis was carried out using FEAT (FMRI Expert Analysis Tool) Version 6.00, part of FSL (FMRIB’s Software Library^1^). Pre-statistics processing included; motion correction using the Linear Motion Correction tool (MCFLIRT); slice-timing correction using Fourier-space time-series phase-shifting for interleaved slice acquisition; non-brain removal using the Brain Extraction tool (BET); spatial smoothing of FWHM 5 mm; grand-mean intensity normalization of the entire 4D dataset by a single multiplicative factor; highpass temporal filtering with a 80 s cutoff. No volumes were deleted as three dummy scans were acquired and discarded during acquisition to allow for signal equilibration. Functional images were registered to high-resolution structural images, which in turn were registered to standard space (MNI152 average image) using the Linear Image Registration tool (FLIRT).

First level/time-series statistical analysis of single subject data was carried out using FMRIB’s Improved Linear Modeling (FILM) with local autocorrelation correction. Three sets of explanatory variables (EVs) were generated based on boxcar functions that corresponded with the visual presentation of the negative, neutral, and positive stimulus blocks. EVs were convoluted with a gamma hemodynamic response function. Standard motion parameters (as estimated by MCFLIRT) were included in the model as confound EVs. *Z* (Gaussianised T/F) statistic images were thresholded using clusters determined by *Z* > 2.3 and a (corrected) cluster significance threshold of *p* < 0.05

Higher-level analysis was carried out using FLAME (FMRIB’s Local Analysis of Mixed Effects). A mixed effects contrast analysis was performed to compare migraine versus control group activation for each emotion category. *Z* (Gaussianised T/F) statistic images were thresholded using clusters determined by *z* > 2.3 and a (corrected) cluster significance threshold of *p* < 0.01 (Worsley, 2001). Significant clusters and their local maxima were identified anatomically using the Harvard-Oxford cortical and subcortical structural atlases (Desikan et al., 2006).

### International Affective Picture System (IAPS) Rating Statistical Analysis

As aforementioned, after the imaging session subjects were reshown the presentation and asked to rate each stimulus block for emotional valence and arousal using the 9-point SAM scale. Each subject’s rating of valence and arousal for each stimulus block were averaged for each emotional type (i.e., positive, neutral, and negative). The mean valence and arousal ratings for each emotional type were compared between migraine patients and controls by an independent sample *t*-test, with *p* < 0.05 taken as statistically significant.

## Results

### Subject Demographics and IAPS Valence and Arousal Ratings

Migraine subjects consisted of 20 females and 3 males, with an average age of 32.9 ± 10.0 years. Controls subjects consisted of 19 females and 4 males, with an average age of 31.3 ± 9.3. Migraine and control subjects did not differ in mean age or group gender composition. Migraineurs averaged 5.4 ± 4.1 headaches per month and they had an average duration of the condition of 15.4 ± 9.2 years.

**FIGURE 2:**
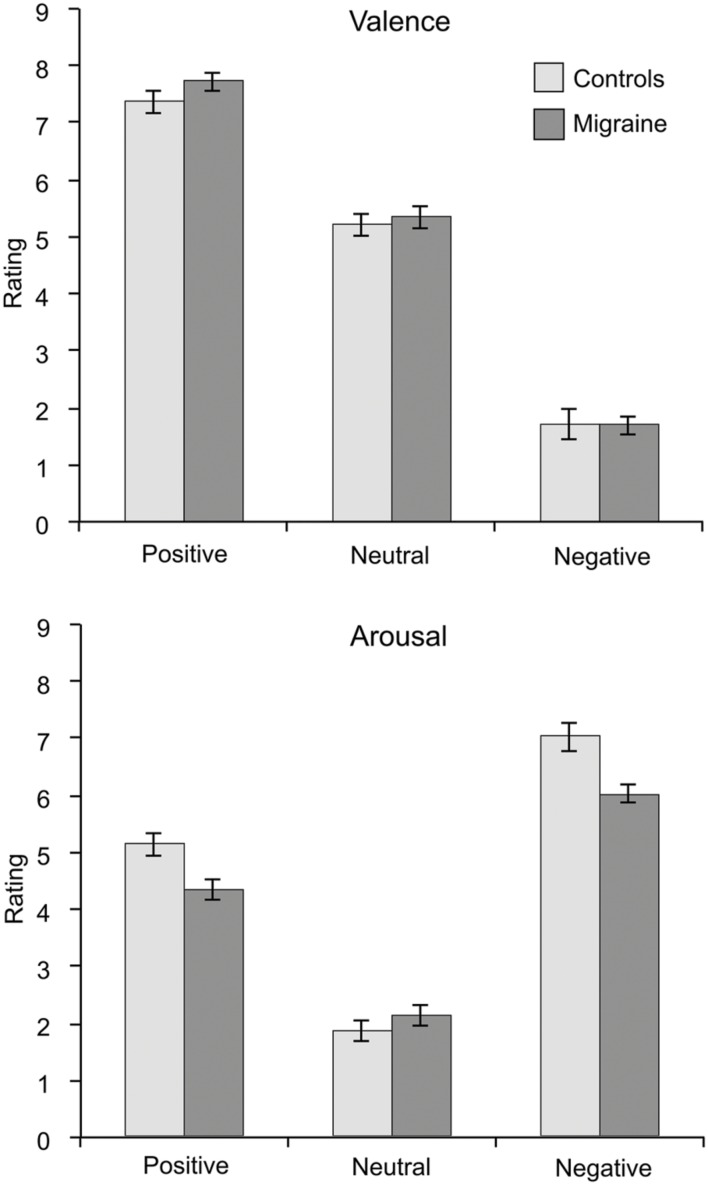
**Mean ratings of valence (upper graph) and arousal (lower graph) for the emotional visual stimuli separated for the migraine and the control groups.** Error bars represent the standard error of the mean. No significant differences were found in any ratings between migraineurs and controls.

Migraine and control subjects did not differ in their ratings of valence or arousal in any emotional category [Positive valence: *t*(44) = 1.415, *p* = 0.164; Positive arousal: *t*(44) = –1.497, *p* = 0.142; Neutral valence: *t*(44) = 0.487, *p* = 0.629; Neutral arousal: *t*(44) = 0.826, *p* = 0.413; Negative valence: *t*(44) = –0.044, *p* = 0.965; Negative arousal: *t*(44) = –1.454, *p* = 0.153]. Mean (and standard error of the mean) ratings for migraine and control groups are shown in **Figure 2**.

### Neural Activation during Emotional Stimuli

Separately, both migraine and control subjects showed similar networks of activated brain regions in response to emotional stimuli (See **Supplementary Figure S1**).

**FIGURE 3:**
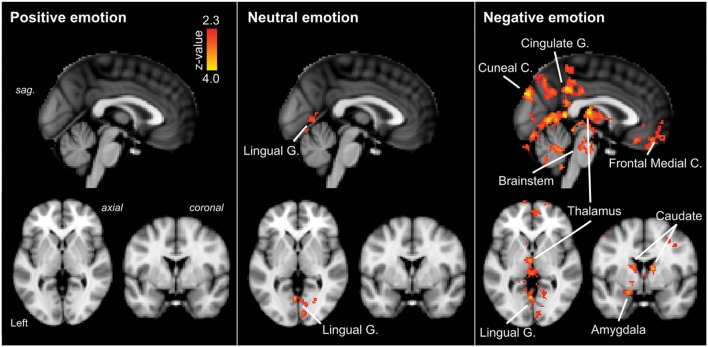
**Regions of significantly increased neural activation in migraineurs, compared to healthy controls, in response to emotional stimuli.** **Left panel**: positive emotional stimuli, **Middle panel**: neutral emotional stimuli, **Right panel**: negative emotional stimuli. The red-yellow color scale represents *Z* values for the contrast of migraineurs greater than controls. Significant clusters are overlaid onto the average MNI152 T1-weighted anatomical brain template. G. – Gyrus, C. – Cortex, sag. – sagittal.

Positive IAPS stimuli: during positive emotional stimuli migraineurs displayed no significant different in neural response, in comparison to controls (**Table 1**; **Figure 3** [Left panel]).

**Table 1:** Regions of significantly different neural activation in migraineurs, compared to healthy controls, during viewing of emotional stimuli.

Brain region	Laterality	*Z*-stat	*P*	MNI coordinates			Voxels
					
				*X* (mm)	*Y* (mm)	*Z* (mm)	
*Positive emotional stimuli:*							
N.A.							
*Neutral emotional stimuli:*							
Migraine>Controls							
Lingual gyrus	R	3.63	0.002	4	-74	-10	550
*Additional Local Maxima*							
Lingual gyrus	L	3.48		- 4	- 66	0	
Intracalcarine cortex	R	3.46		8	-86	2	
Lingual gyrus	R	3.34		14	-76	-6	
Lingual gyrus	R	3.30		24	-70	0	
Lingual gyrus	L	3.19		-2	-68	4	
*Negative emotional stimuli:*							
Migraine>Controls							
Precuneus cortex	L	5.1	3.23e^-26^	-12	-60	50	7919
*Additional local maxima*							
Cuneal cortex	L	5.08		0	- 88	32	
Thalamus (Medial dorsal nucleus)	L	4.69		-4	-14	6	
Lingual gyrus	L	4.5		-2	-64	2	
Precuneus cortex	L	4.45		-14	-60	46	
Precuneus cortex	R	4.31		10	-52	4	
Frontal Medial cortex	R	3.83	1.13e^-06^	2	50	-22	1228
*Additional local maxima*							
Frontal medial cortex/Anterior cingulate	R	3.78		12	34	-14	
Frontal pole	L	3.71		-14	42	-24	
Frontal medial cortex	L	3.61		-12	46	-14	
Frontal pole	R	3.58		8	56	-4	
Frontal medial cortex	R	3.52		4	54	-10	
Frontal pole	R	4.63	3.73e^-05^	22	38	40	895
*Additional local maxima*							
Superior frontal gyrus	R	3.57		22	30	38	
Frontal pole	R	3.56		16	40	40	
Precentral gyrus	R	3.35		38	2	40	
Superior frontal gyrus	R	3.28		26	10	68	
Middle frontal gyrus	R	3.23		40	6	40	
Middle frontal gyrus	L	4.19	1.48e^-04^	-30	22	54	773
*Additional local maxima*							
Middle frontal gyrus	L	4.11		- 30	18	56	
Middle frontal gyrus	L	3.69		-52	12	44	
Middle frontal gyrus	L	3.51		-48	14	40	
Middle frontal gyrus	L	3.38		-42	4	56	
Middle frontal gyrus	L	3.29		-26	32	36	


Neutral IAPS stimuli: during neutral emotional stimuli migraineurs displayed only a single region of increased neural response, in comparison to controls (**Table 1**; **Figure 3** [Middle panel]). This increased activation was observed in visual areas, notably the intracalcarine cortex and lingual gyrus.

**FIGURE 4:**
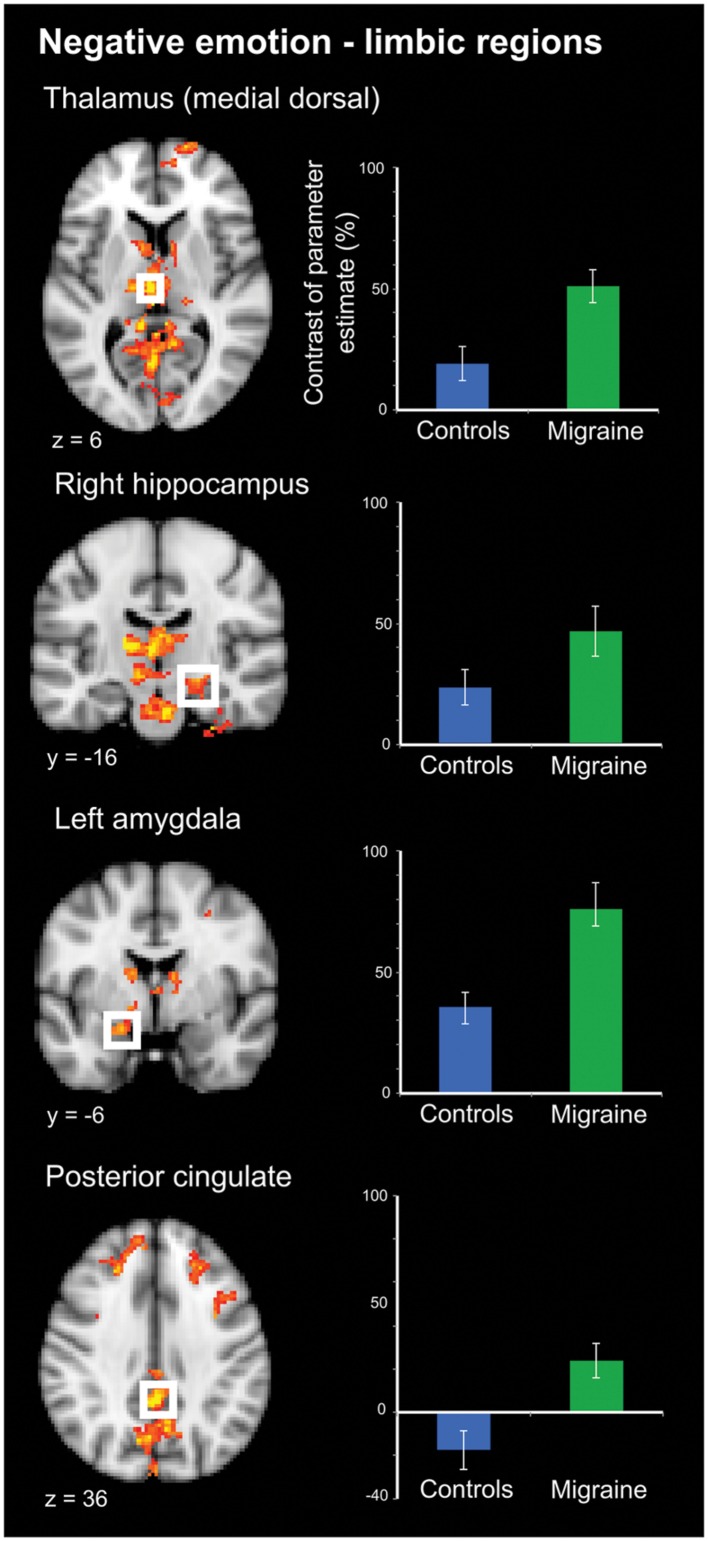
**Limbic regions of significantly increased activation in migraineurs, compared to healthy controls, in response to negative emotional stimuli.** Limbic regions included the medial dorsal thalamus, amygdala, hippocampus, and posterior cingulate. For each region bar graphs of the mean contrast of parameter estimates (COPE; as a percentage) are presented. Error bars represent the standard error of the mean.

Negative IAPS stimuli: during negative emotional stimuli migraineurs displayed a range of regions with increased neural response, in comparison to controls (**Table 1**; **Figure 3** [Right panel] and **Figure 4**). These included increased activation in the superior and middle frontal gyrus, the frontal medial cortex, the frontal pole, posterior cingulate gyrus, precuneus and cuneal cortex, caudate, thalamus, left amygdala and right hippocampus, brainstem (midbrain and pons) and the cerebellum.

## Discussion

This study investigated, for the first time, brain activation associated with emotional processing in episodic migraine during their interictal period. As hypothesized, an increase in neural activity in migraineurs in response to negative IAPS pictures was observed, notably in regions overlapping in their involvement in both nociceptive and emotional processing including the posterior cingulate, caudate, amygdala, and thalamus. These findings support the notion that migraine may feature more generalized altered cerebral processing of aversive/negative stimuli, rather than restricted to sensory stimuli specifically. This more generalized alteration may be an inherent feature of migraine, or may be a consequential alteration produced by the ongoing and repeated nature of migraine. Regardless of its sequela, this more generalized cortical and subcortical increased functional response may form an important part of the migraine pathophysiology and represent an innate sensitivity to aversive stimuli that underpins attack triggers, attack persistence and potentially chronification (Burstein et al., 2015).

### Emotional Appraisal in Migraine

The categories of emotional (positive, neutral, and negative) compared in this study were induced using IAPS (Lang et al., 2008), a standardized affective picture set which has been used extensively in functional neuroimaging studies of emotional processing (de Tommaso et al., 2009; Kamping et al., 2013; Neta et al., 2013; Boucher et al., 2015). Interestingly, ratings of valence and arousal for all three emotional categories did not differ between migraine subjects and healthy controls. In this regard, the increased neural activity in negative emotional processing in migraineurs was not driven by an enhanced conscious emotional impact of the IAPS stimuli. Steppacher et al. (2015) also reported a similar lack of difference in ratings, while in contrast de Tommaso et al. (2009) reported enhanced emotional impact in migraineurs for both positive and negative IAPS stimuli while in concurrent laser-generated pain. In contrast both Steppacher’s and our study were conducted during the interictal (pain-free) state. Taken together this raises the potential that conscious emotional appraisal in migraineurs may be significantly impacted only during a painful state. In support of this theory diary studies of psychological variation in migraineurs over time have shown increased self-report of stress and negative mood directly proceeding and during headache attacks (Harrigan et al., 1984; Sorbi et al., 1996; Spierings et al., 1996). Further study directly contrasting emotional appraisal in the pain and pain-free phases in migraine are needed to clarify this difference and may indeed demonstrate phase-dependent perception and processing of emotional stimuli.

### Central Representation of Pain and Negative Affect

By definition, pain includes both a sensory and affective component (Merskey, 1968; Merskey and Bogduk, 1994). Nociceptive stimuli, including those of trigeminovascular origin involved in headache, activate a wide array of cortical and subcortical areas (Peyron et al., 2000; DaSilva et al., 2002; Apkarian et al., 2005). How activity in these regions gives rise to the complex experience of pain, including both sensory and affective components, remains an area of active discussion (Melzack, 1999; Iannetti and Mouraux, 2010). These areas involved in pain processing share significant spatial overlap with areas involved in processing other domains including emotion, interoception, and reward (Cauda et al., 2012). Interestingly, these areas of overlap were among the regions of increased activity in response to negative emotional stimuli in migraineurs, notably including limbic structures such as the posterior cingulate, amygdala, hippocampus and the (medial dorsal) thalamus (**Figure 4**).

However, the region of increased activation in the cingulate region was ascribed to the posterior cingulate cortex (PCC), which is thought to have little or no involvement in pain processing (in regards to acute pain in healthy subjects). Rather the posterior cingulate has extensive connections with the parietal lobe (which also displayed regions of increased activation) and is proposed to be involved in emotional evaluation including assessment of the self-relevance of emotional stimuli (Vogt, 2005). While the PCC may not be involved in processing physical pain, evidence suggests its involvement in processing psychological pain (including grief and sadness; Meerwijk et al., 2013). Vogt has posited that inactivation of the PCC may be one inherit mechanisms by which the emotional component of pain may be reduced (Vogt, 2005). This raises the possibility that in migraineurs the posterior cingulate may be activated (or insufficiently deactivated) in response to nociceptive input (Aderjan et al., 2010; Maleki et al., 2012b; Russo et al., 2012), with the perceptual outcome a more enhanced or maintained pain perception (Shyu and Vogt, 2009).

The amygdala has long been known for its important role in processing the emotional dimension of pain (Gao et al., 2004; Wiech and Tracey, 2009; Neugebauer, 2015). Among its roles it is thought to modulate motor readiness, autonomic functions, and cognitive processes including attention and memory (Zald, 2003). In the context of pain, the connection between the amygdala and sensory cortical regions (Wiech et al., 2014; Bienvenu et al., 2015) allows emotional arousal to facilitate attention and perception. In the long-term, amygdala-driven somatosensory plasticity (increased cortical representation) has been demonstrated in sensory (auditory) processing (Chavez et al., 2009), suggesting that emotional arousal conjoined with a sensory stimulus may, over time, produce an attentional bias, and/or shift in cortical processing (Wiech and Tracey, 2009). In support of the view that amygdala-sensory plasticity may be an important factor in migraine, several studies have demonstrated altered amygdala connectivity with sensory areas (Hadjikhani et al., 2013; Schwedt et al., 2013), however, this has not been a consensus finding (Hougaard et al., 2015).

### Migraine Models: Expanding Cortical Hyperexcitability and Chronification

Our understanding of migraine pathophysiology has dramatically evolved over the last half-century in increasing acknowledgment that this complex neurological disorder affects multiple cortical, subcortical and brainstem regions that regulate sensory, autonomic, cognitive and affective functions (Burstein et al., 2015). The majority of models of migraine mechanisms have focused on features of the migraine attack itself, including peripheral and central sensitization, cortical hyperexcitability, and cortical spreading. Yet, a complete model of migraine must also account for the more complex emotional milieu of migraine including the prominent role of stress and emotions as attack triggers, prodromal emotional changes, and psychiatric comorbidity. To this end, Maizels et al. (2012) proposed an expansion of the central sensitization model of migraine to include limbic dysfunctions as well as cortical hyperexcitability, a proposal our findings support. This sensitized corticolimbic state would account for the dynamic bidirectional influence of pain and emotion. Limbic regions are also integrated into Burstein and Jakubowski’s (2005) “unitary hypothesis” of migraine triggers and exacerbation, which focuses on limbic and hypothalamic regions. Activity in these regions is influenced by common migraine triggers, including stress and emotional responses, and form part of a parasympathetic pathway for the activation of meningeal nociceptors. These same regions also receive extensive projections from the trigeminovascular nociceptive pathway and are placed to initiate symptoms commonly triggered by headache including stress, fatigue, nausea, and exaggerated emotional responses. This bidirectional network provides a mechanism of afferent and efferent feedback that may drive a migraine attack for many hours and even days (Burstein and Jakubowski, 2005).

As episodic migraine progresses to its chronic form, psychological dysfunction often increases concurrently (Smitherman et al., 2009). Patients with chronic migraine report more somatic complains including fatigue, sleep disturbances and nausea, compared to episodic migraineurs (Maizels and Burchette, 2004). Psychiatric comorbidity, including depression and anxiety, is also increased in chronic migraine (Buse et al., 2013). Migraine progression has been framed as a maladaptation to cumulative stress (allostatic load) over time, including the psychological stress associated with migraine both before and during attacks, as well as interictally (Borsook et al., 2012; Maleki et al., 2012a). In defining allostatic load as the amount of brain activity required to appropriately manage the level of emotional stress at any given time (McEwen and Gianaros, 2011), our results would suggest that even episodic migraineurs display an increased allostatic load in response to negative emotional stress. An avenue of further research would be to compare the response of episodic and chronic migraineurs to emotional stimuli, under the hypothesis that chronic migraineurs may display even greater brain activity as a marker of greater allostatic load. Interestingly, in the context of other pain conditions such as back pain, chronification has been demonstrated to involve a progressive shift away in pain processing from classical sensory regions and more toward engagement of emotional regions (Hashmi et al., 2013). In regards to migraine, determining if such a similar shift is involved in either the early development and/or transformation to chronicity would form a novel and important avenue of research in the evolution of migraine along the lifespan.

### Study Caveats and Future Considerations

While this is the first study to demonstrate increased brain activity in response to general negative emotional stimuli in migraineurs, there are some caveats of our study that should be acknowledged. Though we have interpreted the observed increased activity in migraineurs in response to negative emotional stimuli as evidence of cortico-limbic hyperexcitability we are unable to determine if this represents increased facilitation or dishabituation of emotional processing networks (Coppola et al., 2007; Stankewitz and May, 2007). Additionally, although migraineurs and controls gave equal retrospective ratings of valence and arousal, migraineurs may have experience increased emotion or arousal during the novel presentation and/or reduced emotional habituation over the stimuli period, similar to that demonstrated in response to other stimuli (i.e., visual). By continuously rating during the scanning session, rather than retrospectively, this issue may be clarified in future studies. Additionally, future studies may also include psychophysiological measures, such as engagement of the autonomic nervous system, as additional measures of emotional perception (Bonnet et al., 2015). Furthermore, while emotional stimuli of varying valence (i.e., positive, neutral, and negative) were examined in the current study, future studies may also examine negative emotional stimuli with varying levels of arousal (i.e., high versus low) and salience (i.e., pain-related versus non-pain-related images) to determine if additional factors may also influence this increased central processing.

## Conclusion

Migraine is a multifactorial disorder encompassing not only pain and sensory disturbances but also a complex psychological/affective component active both during attacks and interictaly. A complete model of migraine pathophysiology must integrate all of these diverse components that give rise to the condition (Burstein et al., 2015). Our findings of increased functional activity, namely in cortico-limbic areas including the PCC and amygdala in response to negative emotional stimuli in migraineurs, expands the idea of increased functional activity in response to sensory stimuli to also encompass negative affect. The repeated stress of each migraine attack, and the anticipation of such, results in an ongoing response to an aversive process. Over time, the load of repeated attacks may lead to a more generalized sensitivity to aversive stimuli. An overall enhanced response to negative/aversive stimuli in migraine may underlie the multi/cross sensitization observed in migraine. Equal focus on both the sensory and emotional components of migraine and its pathophysiology may promote a more unified model of migraine that may account for the diversity of triggers, the increased comorbidity with emotional disturbances and the progression from episodic to chronic migraine.

## Author Contributions

SW contributed to the analysis and interpretation of data, drafting of manuscript and critical revision; RV and SS contributed to the acquisition of data; JL contributed to the analysis of data; DH contributed to critical revision; RB contributed to study conception and design; LB contributed to study conception and design, analysis of data and critical revision and DB contributed to study conception and design and critical revision.

## Conflict of Interest Statement

The authors declare that the research was conducted in the absence of any commercial or financial relationships that could be construed as a potential conflict of interest.

## Footnotes

1 www.fmrib.ox.ac.uk/fsl

## Abbreviations

EV: explanatory variable
fMRI: functional magnetic resonance imaging
IAPS: International Affective Picture System
PCC: posterior cingulate cortex
SAM: Self-assessment manikin
