# Supplementary material for: Increased Functional Activation of Limbic Brain Regions during Negative Emotional Processing in Migraine
Source: Front Hum Neurosci. 2016 Jul 26;10:366. doi: 10.3389/fnhum.2016.00366 (PMC4960233; doi:10.3389/fnhum.2016.00366)
Supplement: Supplementary file 1 [file Table_1.DOCX]

Supplemental Table 1. Clinical characteristics (including medication usage) for individual migraine subjects.

| **Subject ID** | **Migraine Frequency/month** | **Migraine**  **Duration** | **Disease duration** | **Visual Aura** | **Photophobia** | **Medications for Migraine** | **Medications not for Migraine** |
| --- | --- | --- | --- | --- | --- | --- | --- |
| M01 | 2 | n.a. | 20 | Yes | Yes | None | None |
| M02 | 15 | 8-48 | 14 | No | Yes | None | None |
| M03 | 6 | 7-8 | 8 | Yes | Yes | Rizatriptan, amitriptyline, atenolol | Nuvaring, Levoxyl, Liothyronine, Atenolol, Amitryptyline, Flonase |
| M04 | 12 | 10 | 7 | No | Yes | Ibuprofen, acetaminophen | Zarah, Minocyclin |
| M05 | 1 | 8-72 | 3 | Yes | Yes | Caffeine+acetaminophen | Naproxen |
| M06 | 2 | 5-7 | 21 | Yes | Yes | Ibuprofen | None |
| M07 | 3 | 24 | 23 | No | Yes | Maxalt, acetaminophen | Colazal, Levothyroxine, Remicade |
| M08 | 9 | 24 | 3 | No | Yes | None | None |
| M09 | 7 | 72 | 28 | Yes | Yes | Ketorolac, darvocet* | Oral contraceptives, multivitamin, probiotics, magnesium, borage, vitaminD, vitamin E, Wellbutrin*, Vyvanse* |
| M10 | 2 | 24-72 | 9 | No | Yes | Naratriptan* | Celexa, Wellbutrin |
| M11 | 4 | n.a. | 31 | Yes | Yes | Sumatriptan, butalbital | Levothyroxine, multivitamin |
| M12 | 3 | 48 | 15 | Yes | Yes | Topiramate, sumatriptan | Calcium, AREDs eye supplement, Valtrex, vitamin D |
| M13 | 8 | 24-72 | 27 | Yes | Yes | Topiramate, sumatriptan, tramadol, ibuprofen | Zyrtec |
| M14 | 2 | 24 | 12 | No | Yes | Sumatriptan, naproxen | Seasonique, Benadryl, magnesium*, multivitamin* |
| M15 | 3 | 72-96 | 26 | Yes | Yes | None | Atorvastatin, Ambien* |
| M16 | 8 | 8 | 14 | No | Yes | None | None |
| M17 | 2 | 36 | 23 | No | Yes | Ibuprofen | None |
| M18 | 10 | 3-6 | 7 | No | Yes | Acetaminophen, ibuprofen | None |
| M19 | 1 | 8 | 5 | Yes | Yes | None | Oral contraceptive |
| M20 | 4 | 10 | 15 | No | Yes | Ibuprofen | None |
| M21 | 4 | 10 | 30 | Yes | Yes | Ibuprofen, excedrin | None |
| M22 | 4 | 4-12 | 12 | No | Yes | None | None |
| M23 | 14 | 8-30 | 3 | Yes | Yes | None | Oral contraceptive |

* Taken as needed

n.a. – not available
