# Supplementary material for: Increased Functional Activation of Limbic Brain Regions during Negative Emotional Processing in Migraine
Source: Front Hum Neurosci. 2016 Jul 26;10:366. doi: 10.3389/fnhum.2016.00366 (PMC4960233; doi:10.3389/fnhum.2016.00366)
Supplement: Supplementary file 2 [file Image_1.PDF]

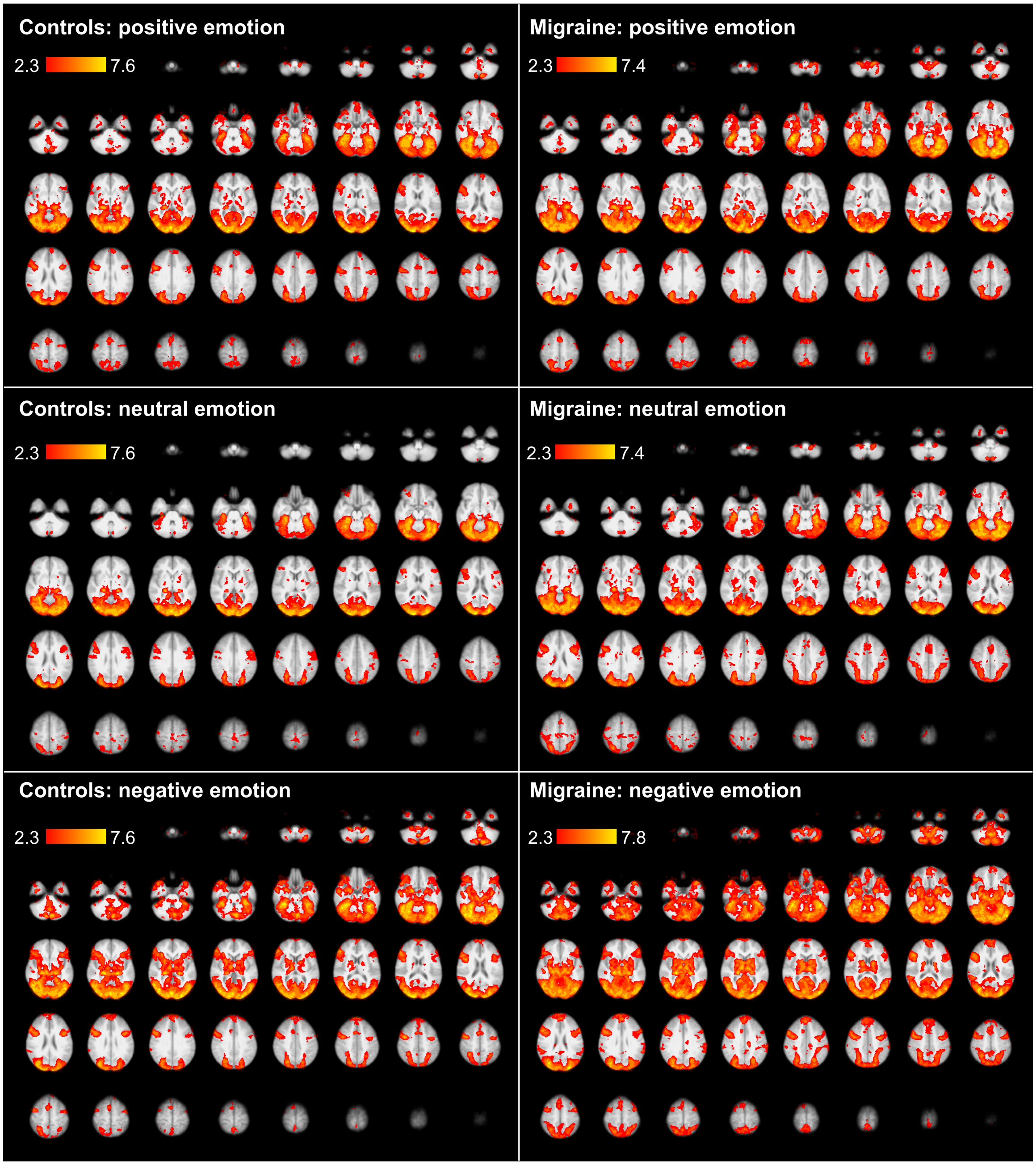

Supplemental Figure 1. Average group activation maps for migraine patients and controls for the three emotional stimuli (Top panel: positive, Middle panel: neutral and Lower panel: negative). FMRI data processing was carried out using FEAT (FMRI Expert Analysis Tool) Version 6.00, part of FSL (FMRIB's Software Library, www.fmrib.ox.ac.uk/fsl). Z (Gaussianised T/F) statistic images were thresholded using clusters determined by Z>2.3 and a (corrected) cluster significance threshold of p<0.05.
